# Ameloblastic Fibroma of the Mandible Reconstructed with Autogenous Parietal Bone: Report of a Case and Literature Review

**DOI:** 10.1155/2019/5149219

**Published:** 2019-06-18

**Authors:** Conor Carroll, Mishaal Gill, Eleanor Bowden, John Ed O'Connell, Rajeev Shukla, Chris Sweet

**Affiliations:** ^1^Department of Oral and Maxillofacial Surgery, Alder Hey Children's NHS Foundation Trust, East Prescot Road, Liverpool L14 5AB, UK; ^2^Department of Pathology, Alder Hey Children's NHS Foundation Trust, East Prescot Road, Liverpool L14 5AB, UK

## Abstract

Ameloblastic fibroma (AF) is a rare, slow-growing benign neoplasm, comprised of tissues of odontogenic origin. It constitutes 2% of odontogenic tumours, occurring at any age, but has a predilection to present in the first two decades of life. AF principally affects the posterior mandible. It is characterized by epithelial islands and cords immersed in ectomesenchyme that mimics the dental papilla and enamel organ but without actual hard tissue formation. Herein, we describe the case of a 6-year-old Caucasian male who presented to the Oral and Maxillofacial Department at Alder Hey Children's Hospital, Liverpool, UK, with a painless expansile mass in the left mandible which was diagnosed as a benign ameloblastic fibroma and subsequently enucleated and reconstructed with a parietal calvarial bone graft. A brief literature review and the issues surrounding diagnosis are discussed.

## 1. Introduction

Ameloblastic fibroma (AF) is a tumour, classified under the World Health Organization as “odontogenic epithelium with odontogenic ectomesenchyme, with or without hard tissue formation” [1]. They tend to develop “de novo,” without an apparent aetiological factor. They are usually asymptomatic and may be identified incidentally as a radiographic finding during routine examination. They are benign in nature and share some clinical, radiographic, and histological features similar to other mixed odontogenic tumours, for example, ameloblastic fibro-odontoma (AFO), ameloblastic fibrodentinoma (AFD), complex and compound odontoma, odontoameloblastoma, and calcifying odontogenic cyst. These lesions can pose a diagnostic and therapeutic challenge.

We present a rare case of AF affecting the left body of mandible in a 6-year-old boy, which was surgically enucleated and reconstructed with a parietal calvarial bone graft (CBG).

## 2. Case Presentation

A 6-year-old boy was referred to the Oral and Maxillofacial Department at Alder Hey Children's Hospital, Liverpool, UK, with a painless left mandibular swelling. The mass had been present for two weeks and was gradually increasing in size. There was no complaint of difficulty in mastication, and there was no history of paraesthesia or discharge. The patient was systemically well and a full blood count was within normal limits. He had no relevant medical, drug, or familial history.

**Figure 1:**
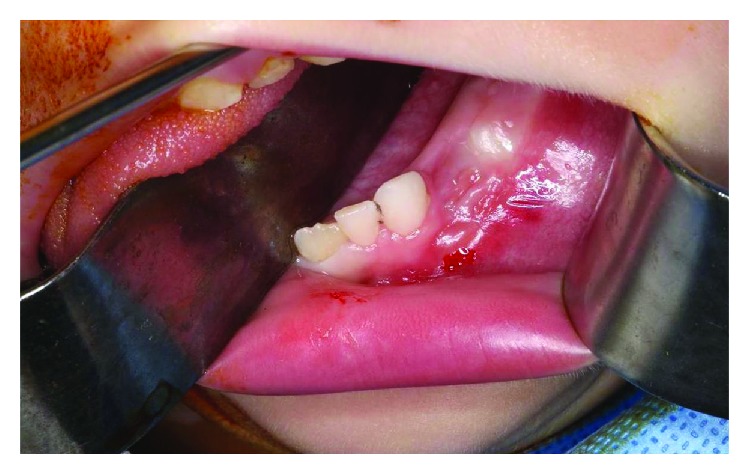
Intraoral view demonstrating swelling overlying the alveolar ridge with associated expansion of the left buccal plate. (Note that the lower left first and second deciduous molars were extracted previously at the incisional biopsy).

Clinical examination did not reveal any facial asymmetry or cervical lymphadenopathy. Intraorally, there was a localized swelling with expansion of the mandibular buccal plate extending from the left mandibular canine to the left first permanent molar (Figure 1). On palpation, the swelling was nontender with a hard consistency and was fixed to the deeper tissues. The overlying mucosa was within normal limits. All four first and second deciduous molars were carious, and the lower left first deciduous molar and lower left second deciduous molar were splayed due to the lesion.

**Figure 2:**
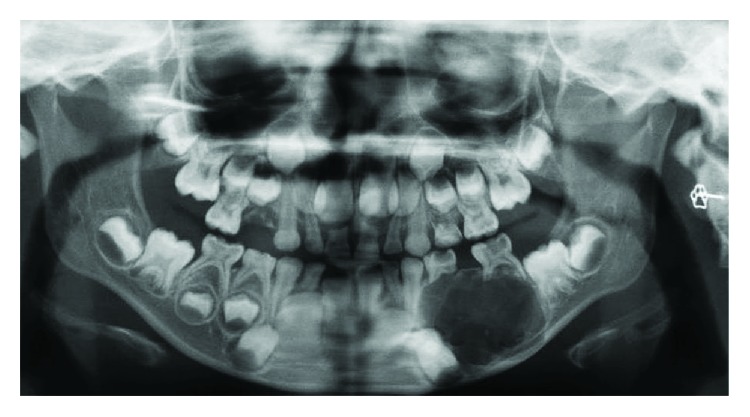
Orthopantomogram showing a well-defined multilocular radiolucent lesion with a sclerotic border in the left body of the mandible. The second deciduous molar has been displaced distally and is supraerupted.

**Figure 3:**
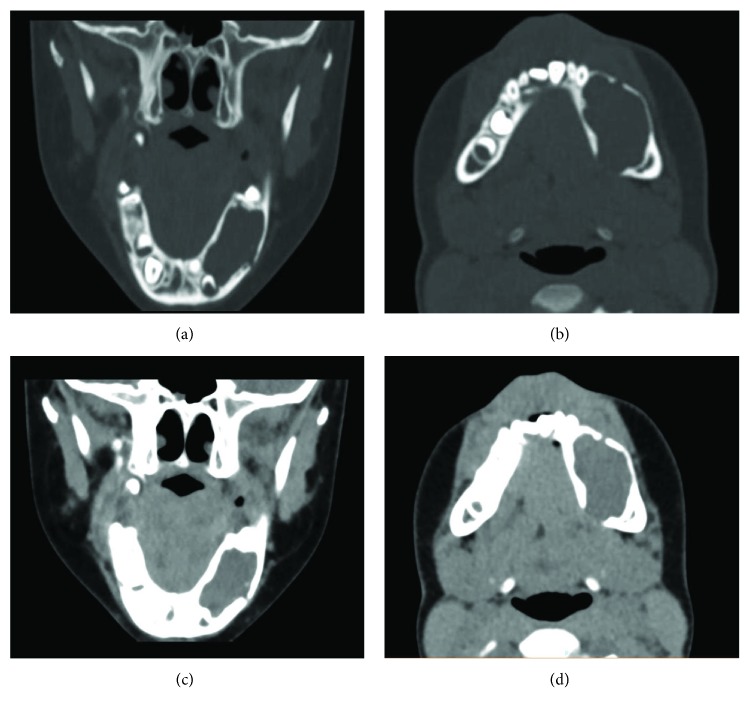
(a–d) Computed tomogram demonstrates an expansile mass to the left mandible with small buccal and lingual perforation of the cortices.

**Figure 4:**
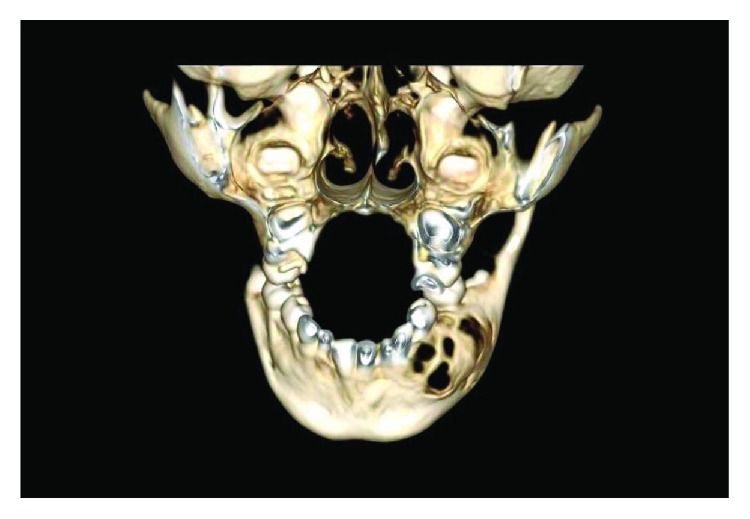
Three-dimensional tomographic reconstruction illustrating bony destruction with fenestration.

Radiographically, an orthopantomogram (OPG) showed a multilocular, radiolucent lesion with scalloped margins affecting the left hemimandible (Figure 2). It extended anteroposteriorly from the distal aspect of the unerupted lower left canine to the mesial aspect of the lower left first permanent molar and approached the inferior border of mandible. The roots of the lower left first and second deciduous molars were resorbed, and the first and second premolar tooth germs were absent. A computed tomogram (CT) showed marked cortical thinning and some internal calcification but no evidence of internal septations. In some areas, the cortex appeared breached (Figures 3(a)–3(d) and 4).

**Figure 5:**
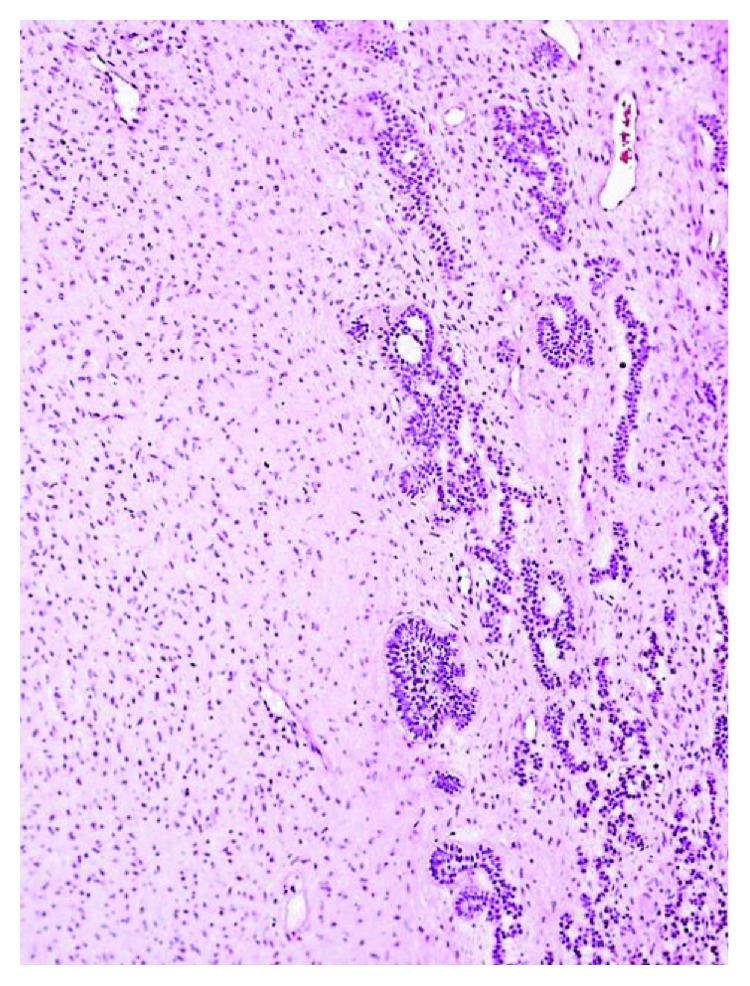
H&E (×100) biphasic lesion with dominant stromal and smaller epithelial component.

**Figure 6:**
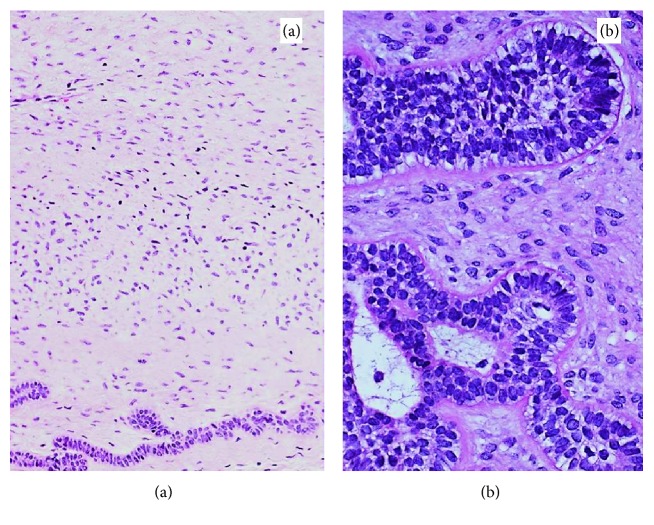
(a) H&E (×200) stromal element is composed of bland spindle cells with no cellular atypia or mitosis; small islands and cords of markedly attenuated ameloblastic epithelium are seen at the bottom of the field. (b) H&E (×400) epithelial element with peripheral palisading and reverse polarization away from basement membrane (Vickers-Gorlin change).

An incisional biopsy and removal of all carious primary teeth under general anaesthesia was performed. The lesion was submitted for histopathological examination. Histological sections (Figures 5, 6(a), and 6(b)) showed a soft tissue specimen consisting of cellular/fibroblastic fibromyxoid stroma resembling primitive mesenchyme or developing dental papillae. The stromal fibroblasts had a diffuse or nodular arrangement. Towards the periphery of the fibroblastic stroma were various collections of “budding” cords and nests of odontogenic epithelium. Some islands of cells showed peripheral palisading and central squamatization and calcification. Several of the cords were rimmed by hyalinised material but not developed enough to qualify as dentine. Given that the lesion appeared uniformly radiolucent on imaging and did not include aberrant tooth germ-like structures, the features were consistent with a diagnosis of an AF.

**Figure 7:**
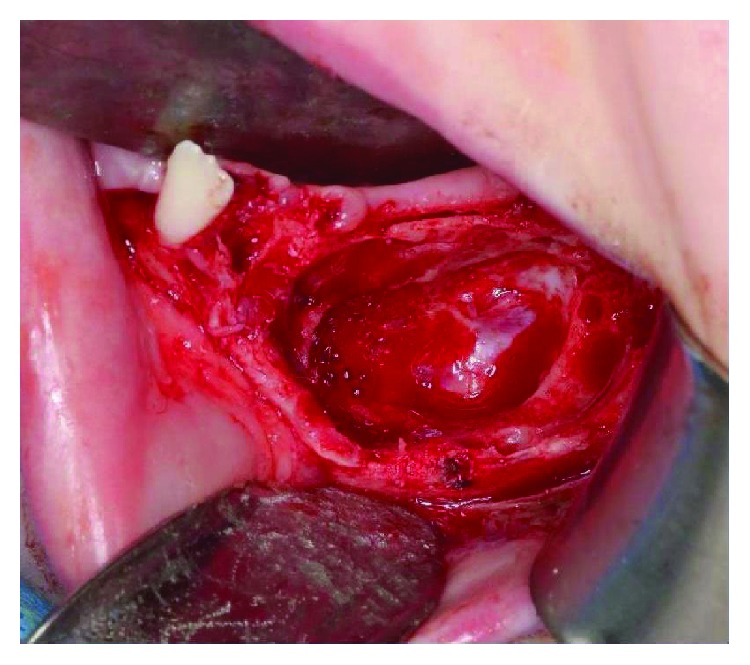
The tumour cavity in body of mandible following enucleation and curettage.

The case was discussed at a craniofacial multidisciplinary team meeting. The proposed treatment involved enucleation of the tumour and reconstruction of the defect with a full-thickness parietal calvarial bone graft. The lesion was exposed via a transoral mucoperiosteal flap which extended from the lower left central incisor to the lower left wisdom tooth. While the lesion was successfully enucleated (Figure 7), it was found to have perforated both the buccal and lingual (posterosuperiorly adjacent to the molar teeth) cortices. In addition, it was found to be enveloping the mental nerve via the foramen and therefore the nerve was sacrificed.

The parietal bone graft was harvested via a full-thickness parietal scalp incision. The outer and inner tables were harvested as a single block, then separated on a “back-table,” with the inner table replaced over the dura and secured via titanium miniplates. The outer cortex and underlying cancellous bone were then cut into several small pieces (cortic-cancellous) facilitating placement into the mandibular defect.

**Figure 8:**
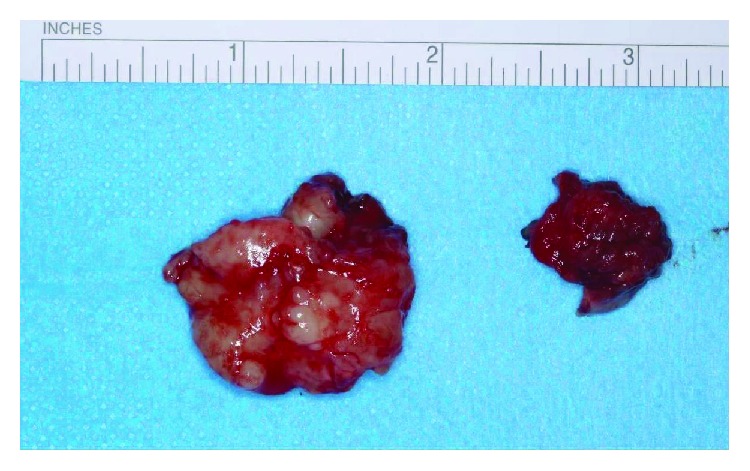
Gross pathological specimen.

The surgical specimen was then sent for formal histopathological examination. Gross pathological analysis showed an irregular mass of white rubbery tissue measuring 30 × 25 × 15 mm (Figure 8). The cut surface was yellowish/white in colour with a rubbery consistency and clearly extended to the resection margin of the enucleation. Histological sections showed small islands and strands of basophilic ameloblastic epithelium in a background of abundant stroma with bland oval to spindle cells. No necrosis, atypia, or mitoses were present. There was focal inflammation and collection of macrophages. Further soft tissue specimens from the superior, inferior, and posterior-lingual margins did not show any tumour invasion. Overall, the histological features were considered to be characteristic of a conventional ameloblastic fibroma, neither the granular nor cystic variant.

**Figure 9:**
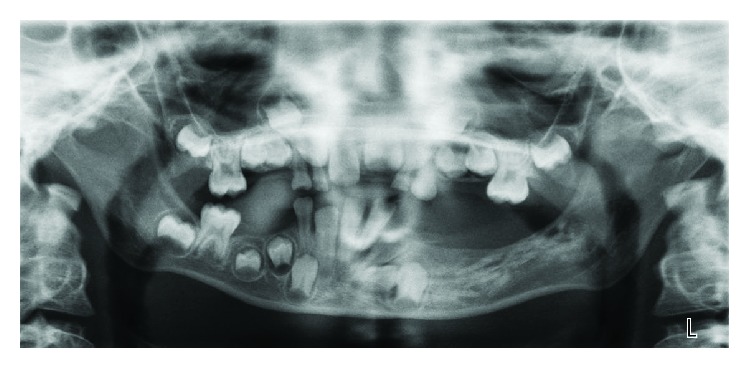
Postoperative OPG demonstrating satisfactory healing in the left body of the mandible with no evidence of recurrence.

The postoperative period was uneventful, and the patient was discharged two days following surgery. He is currently under regular clinical and radiographic follow-up. An OPG taken 10 months post-surgery shows good bone formation with no signs of tumour recurrence (Figure 9).

## 3. Discussion

An electronic literature search was conducted using the PubMed, Embase, and CINAHL applications. The general search criteria were (Ameloblastic)∗, (Fibroma)∗, (Ameloblastic Fibroma)∗, and (Odontogenic Tumor)∗. In the second phase of the review, terms related to the initial keywords used above were enabled to allow for a broader search criteria of the topic. This allowed for wider search terms across all databases to include all articles which may be relevant. The literature review yielded 604 papers; duplicates were then removed to provide 334 papers.

Evidence of greatest hierarchical value was a systematic review on AF by Chrcanovic et al., 2017; the most common publications that are related to AF and ameloblastic fibrosarcoma (AFS) were case reports and literature reviews descriptive in nature.

The first publication of AF was by Kruse in 1891 [2]. It is considered to be the least differentiated of the group of mixed odontogenic tumours as the neoplastic cells do not produce calcified tooth tissue, i.e., enamel and dentine [3]. AF is a true mixed odontogenic tumour as both the epithelial and ectomesenchymal tissues are neoplastic [4].

It is reported that AF has a propensity to affect males more than females in a ratio of 1.4 : 1 [4]. It has a predilection to occur in the first and second decades of life [5], although cases have been reported in middle-aged groups, for example, that of an extensive AF in a 45-year-old male [6]. The most commonly affected site is the posterior mandible [7]. There have been reported cases arising in the maxillary sinus [8].

The most likely presentation is that of a unilateral painless swelling [4, 5]. Associated characteristics are mobility of teeth, root resorption, expansion of buccal and lingual cortices, pathological fracture, and paraesthesia [9, 10]. The lesion may be mistaken to be a dentigerous cyst as it can be associated with delayed/failure of tooth eruption [4, 11–13].

The radiographic features are variable, ranging from a well-circumscribed small lucent unilocular lesion to a more expansile multiloculated appearance seen in larger tumours [14]. The borders of the lesion are well-defined with sclerotic margins [7]. There may be cortical expansion in a buccolingual plane but this may be misinterpreted on a 2D image and therefore, we advocate the use of computed tomography to assess tumour extent and invasion. Furthermore, in cases where soft tissue invasion is suspected, magnetic resonance imaging should be considered.

Histologically, AF is a biphasic tumour made up of odontogenic ectomesenchyme resembling tooth-related structures such as dental papilla and epithelial strands and nests similar to the dental lamina and enamel organ, but without dental hard tissues [15]. The stromal component features spindled and angular cells with little collagen, imparting a myxomatous appearance. The epithelial component is made up of thin cords or small nests of odontogenic epithelium with little cytoplasm and basophilic nuclei.

Histological differential diagnosis of AF includes its malignant counterpart AFS and ACS and other mixed odontogenic tumours. AF along with AFD and AFO histologically resemble various stages of odontogenesis. AF lacks significant dental hard tissue formation such as dentin or enamel. If there is dentin or enamel present, the lesion is classified as AFD or AFO, respectively [16]. AFS is a neoplasm with a similar architecture to AF, but it is composed of a benign epithelium and malignant mesenchymal tissue typically comprising marked cellularity, nuclear pleomorphism, and a moderate-to-high number of mitotic figures in the mesodermal component. These characteristic histological findings were absent in this case which supports the diagnosis of AF.

In general terms, AF is primarily diagnosed morphologically, with immunohistochemistry having a limited diagnostic role. Ki-67, p53, and proliferating cell nuclear antigen (PCNA) are useful biomarkers of malignant transformation of AF into AFS in borderline cases, as AFS shows higher positivity of these markers [17, 18].

Immunohistochemistry has been applied to understand histogenesis. AF express CK7, CK13, and CK14, similar to the immunophenotype of the dental lamina [19]. A recent study assessed the immunohistochemical expression of odontogenic ameloblast-associated proteins; amelotin, ameloblastin, and amelogenin in diverse odontogenic tumours, including AF. Among these four proteins, AF was positive only for amelogenin. This further supports that the tumour cells of AF recapitulate dental lamina cells [20].

The management of AF can be a challenge, and there is no clear consensus regarding the optimal approach. In our opinion, a “case-specific” approach is appropriate. The aims of treatment are to remove the tumour and decrease the chances of recurrence while preserving adjacent vital structures. Management is dictated by patient age, extent and spread of the lesion, and histopathological findings.

In young patients, an AF could represent the primitive stages of a developing complex odontoma [4, 21]. Currently, we are unable to differentiate a hamartomatous lesion from a neoplasm merely on histological grounds; thus, age of the patient should be a significant factor when choosing therapeutic management.

Philipsen et al. [22] proposed that the innocuous behaviour of the lesion does not justify aggressive initial treatment but rather meticulous surgical enucleation with close clinical follow-up.

This is especially pertinent in a young patient where the emphasis is to preserve masticatory function and maintain dentofacial growth.

A more radical approach of marginal or segmental resection is suggested by some authors because of the possibility of malignant transformation of an ameloblastic fibroma [23, 24]. It is thought that around one-third of ameloblastic fibrosarcomas develop as a result of the malignant transformation of an ameloblastic fibroma [25]. Most papers included in our literature review agree a conservative surgical approach initially, followed by further aggressive excision for recurrent lesions, very large tumours, or those involving the maxilla.

In a large review of 123 cases of AF by Chen et al. [26], univariate analysis of malignant transformation-free survival indicated that, among all the analysed clinical variables, only the age of patients at the first presentation was significantly related to malignant transformation of AF. Patients younger than 22 years were unlikely to develop malignant transformation (3.3%) in comparison to patients older than 22 years (26.1%).

There is conflicting data in the literature on the recurrence rate of AF. Furthermore, not all reported cases have long-term follow-up, and so it is difficult to determine the prognosis of AF. Dallera et al. [21] reported no recurrences for 5 cases treated with enucleation and curettage with an average follow-up period of 15 years. A similar review of 9 AF cases by Gorlin et al. [8] indicated that there was no recurrence subsequent to conservative therapy. However, in a review of the literature on recurrences of AF, Zallen et al. [27] found a cumulative recurrence rate of 18.3%. The reason for the discrepancy in recurrence rates is uncertain and suggests that the cause of recurrence is due to incomplete removal and presence of satellite tumours at the edge of the lesion [6].

Long-term follow-up is recommended [28]. It is our intention to review our patient (clinically and radiographically) at 3 monthly intervals, for 6 months, followed by 6 monthly intervals for 2 years, and yearly thereafter for a prolonged period of time, likely in the region of 10-15 years given that malignant transformation can occur years after initial diagnosis.

In our opinion, reconstructive options should be case and defect-specific and depend on a number of factors including site and extent of defect, patient age, and associated medical comorbities. Autogenous bone grafts are considered to be the gold standard for maxillofacial reconstruction [29]. In this particular case, the use of a parietal calvarial bone graft to reconstruct the mandibular defect was utilised. We chose to reconstruct immediately given the distinct histological findings from the initial biopsy and to avoid exposing the patient to a repeat general anaesthetic. Given the extent of the defect and loss of integrity of the buccal and lingual cortical plates, reconstruction with autogenous parietal bone provided structural stability to the mandible and aided against pathological fracture. Furthermore, osteogenesis will facilitate adequate bone formation to assist oral rehabilitation with strategic placement of dental implants when the patient is older [30, 31].

Calvarial bone grafts are considered to be a safe and effective source of bone due to their low donor and recipient site complications [32]. This is because of the greater potential for osteointegration and revascularization as the cortical bone can act as a rigid platform for the regeneration of new tissue. The volume of bone harvested to reconstruct a large defect may be a limiting factor to this technique. However, in this case, given the size of the mandibular defect to be restored, this was not significant. The possible complications which may be encountered when harvesting a calvarial bone graft include dural tear, intracerebral haematoma, cerebrospinal fluid leaks, meningitis, and aesthetic defects to the skull. In a study of fifty consecutive patients treated with a CBG for maxillofacial reconstruction, a low donor and recipient site complication rate of 4 and 4.8%, respectively, was found [33].

The CBG is considered a viable alternative to the anterior iliac crest graft [32]. However, given that our patient was six years of age, this donor site was not chosen for mandibular reconstruction because of: incomplete ossification of the growth plates; to avoid damage to the lateral cutaneous nerve and; to avoid disturbance to iliac wing growth [34]. Major complications of iliac crest bone grafting can also include chronic pain, arterial injury, arteriovenous fistula formation, abdominal organ herniation, and pelvic instability [34].

In extensive cases partial mandibular resection may be indicated involving reconstruction with rigid fixation or a composite vascularised free-flap.

## 4. Conclusion

This case demonstrates that ameloblastic fibroma can be managed with enucleation and immediate reconstruction with autogenous parietal bone. Patients with AF, however, must be followed up for a long period because of AF's ability to transform into its malignant counterpart, ameloblastic fibrosarcoma. Heretofore, there has been no clinical or radiographic evidence for recurrence 2 years postoperatively, and the patient has made a successful return to function.

## Abbreviations

AF: Ameloblastic fibroma
AFS: Ameloblastic fibrosarcoma
AFD: Ameloblastic fibrodentinoma
AFO: Ameloblastic fibro-odontoma
ACS: Ameloblastic carcinosarcoma
CBG: Calvarial bone graft.
